# Activin A, a Novel Chemokine, Induces Mouse NK Cell Migration via AKT and Calcium Signaling

**DOI:** 10.3390/cells13090728

**Published:** 2024-04-23

**Authors:** Yunfeng Wang, Zhonghui Liu, Yan Qi, Jiandong Wu, Boyang Liu, Xueling Cui

**Affiliations:** 1 Department of Immunology, College of Basic Medical Sciences, Jilin University, Changchun 130021, China; yunfeng19@mails.jlu.edu.cn (Y.W.); liuzh@jlu.edu.cn (Z.L.); qiyan@jlu.edu.cn (Y.Q.); 2 Key Laboratory of Neuroimmunology and Clinical Immunology, Changchun 130021, China; 3 Bionic Sensing and Intelligence Center, Institute of Biomedical and Health Engineering, Shenzhen Institute of Advanced Technology, Chinese Academy of Sciences, Shenzhen 518055, China; jd.wu@siat.ac.cn; 4 Department of Genetics, College of Basic Medical Sciences, Jilin University, Changchun 130021, China; 5 Department of Scientific Research, Jilin Jianzhu University, Changchun 130118, China

**Keywords:** natural killer cells, migration, activin A, AKT signaling, calcium signaling

## Abstract

Natural killer (NK) cells can migrate quickly to the tumor site to exert cytotoxic effects on tumors, and some chemokines, including CXCL8, CXCL10 or and CXCL12, can regulate the migration of NK cells. Activin A, a member of the transforming growth factor β (TGF-β) superfamily, is highly expressed in tumor tissues and involved in tumor development and immune cell activation. In this study, we focus on the effects of activin A on NK cell migration. In vitro, activin A induced NK cell migration and invasion, promoted cell polarization and inhibited cell adhesion. Moreover, activin A increased Ca^2+^, p-SMAD3 and p-AKT levels in NK cells. An AKT inhibitor and Ca^2+^ chelator partially blocked activin A-induced NK cell migration. In vivo, exogenous activin A increased tumor-infiltrating NK cells in NS-1 cell solid tumors and inhibited tumor growth, and blocking endogenous activin A with anti-activin A antibody reduced tumor-infiltrating NK cells in 4T-1 cell solid tumors. These results suggest that activin A induces NK cell migration through AKT signaling and calcium signaling and may enhance the antitumor effect of NK cells by increasing tumor-infiltrating NK cells.

## 1. Introduction

Natural killer (NK) cells, which are classified as innate lymphoid cells (ILCs), are widely distributed in lymphoid and non-lymphoid organs and tissues, such as peripheral blood, spleen, lymph nodes, liver and lung [1,2,3]. Most murine NK cells express CD49b and NKp46, while the NK1.1 epitope is expressed in C57BL/6 mice. NK cells in peripheral blood are recruited by environmental factors, such as infection and tumor microenvironment, and can rapidly migrate to local tissues [4,5]. NK cells serve as the first line of the innate immune defense system, which can directly kill tumor cells without prior antigen stimulation and activation [6,7]. Moreover, recent studies have reported that NK cells have the ability to regulate tumor growth by sensing specific growth factors; for example, platelet-derived growth factor (PDGF) produced in many tumors and its receptor-β (PDGFRβ) signaling can enhance IL-15-mediated NK cell survival, and PDGF-DD engagement of NKp44 triggers NK cell secretion of IFN-γ and TNF-α resulting in the arrest of tumor cell growth [7,8]. This highlights the importance of various cytokines in modulating the antitumor effects of NK cells, with the chemotaxis of NK cells to tumor sites playing a crucial role in initiating NK-cell-mediated antitumor responses.

Activin A is a growth and differentiation factor that is a member of the transforming growth factor β (TGF-β) superfamily [9,10] and plays an important role in tumorigenesis and tumor development. Similarly to other members of the TGF-β superfamily, activin A binds to cell surface serine/threonine kinase receptors, namely the type II activin receptor (ActR II) and the type I activin receptor (ActR I), and then transmits signals through the canonical SMAD signaling pathway or the non-SMAD signaling pathway to regulate target gene transcription [11,12]. Recent studies have reported that various immune cells can synthesize and secrete activin A, such as monocytes/macrophages, neutrophils, mast cells and NK cells [9,10,13,14]. Additionally, activin A plays a crucial role in regulating the activities of various immune cells, including T cells, B cells, NK cells, monocytes/macrophages, neutrophils and microglia [14,15,16,17,18]. Activin A in the tumor microenvironment may affect tumors’ development by regulating immune cells’ function.

Many factors influence the migration and infiltration of NK cells. For example, CXCL12 can bind to the CXC chemokine receptor 4 (CXCR4) on the surface of NK cells and induce NK cell chemotaxis [19]. Recent studies have reported that activin A can induce the migration of fibroblasts and microglia as well as inhibit neutrophil migration induced by fMLP [16,20,21], suggesting that activin A exhibits different regulatory effects on the migration of different cells. Nevertheless, it remains unclear whether activin A can induce NK cell migration and affect NK cell tumor infiltration. In this study, we used microfluidic chips and transwell chambers to determine the effect of activin A on mouse NK cell migration. Furthermore, we studied NK cell infiltration through tumor-bearing experiments. These results indicate that activin A acts as a novel chemokine to enhance mouse NK cell infiltration into tumors.

## 2. Materials and Methods

### 2.1. Ethics Statement

All animal studies were adhered to the Principles of Laboratory Animal Care and conducted in accordance with the guidelines and approval by Jilin University. A laboratory animal ethics review form was approved by the Laboratory Animal Ethics Committee of the College of Basic Medical Sciences of Jilin University (No. 2021-213).

### 2.2. Reagents

Recombinant human/mouse/rat activin A (Cat# 338-AC), anti-human/mouse/rat activin A beta A subunit antibody (Cat# AF338) and enzyme-linked immunosorbent assay (ELISA) kit for activin A (Cat# DAC00B) were provided by R&D systems (Minneapolis, MN, USA). Recombinant murine CXCL12 (SDF-1α, Cat# 250-20A) and recombinant murine IL-2 (Cat# 212-12) were obtained from PeproTech (Rocky Hill, NJ, USA). Giemsa (Cat# 32884), phalloidin (Cat# P5282) and DMSO (Cat# D2650) were purchased from Sigma-Aldrich (Merck KGaA, Darmstadt, Germany). Fibronectin (Cat# ab92784) was purchased from Abcam (Cambridge, UK). Cell Counting Kit-8 (CCK-8, Cat# GK10001) was bought from GlpBio Biotechnology Co., Ltd. (Shanghai, China). PI (Cat# ST511) and DAPI (Cat# C1002) were purchased from Byeotime (Shanghai, China). Matrigel (Cat# 356234) was obtained from Corning (New York, NY, USA). The antibodies to mouse CD49b-PE (Cat# 553858) and NKp46-FITC (Cat# 560756) were provided by BD Biosciences (San Jose, CA, USA). The polyclonal rabbit anti-GAPDH antibody (Cat# abs132004) and AKT inhibitor (GSK690693, Cat# abs812028) were bought from Absin (Shanghai, China). Mouse monoclonal antibody against β-catenin (Cat# M24002) was obtained from Abmart (Shanghai, China). Rabbit polyclonal antibodies against vimentin (Cat# bs-0756R) were obtained from Bioss (Beijing, China). Rabbit polyclonal antibodies against MMP2 (Cat# A6247), phospho-Smad3-S423/S425 (p-Smad3, Cat# AP1263) and Smad3 (Cat# A22133) were obtained from ABclonal (Wuhan, China). Rabbit monoclonal antibodies against phospho-AKT (p-AKT, Cat# 4060) and AKT (Cat# 4691) were provided by Cell Signaling Technology (Danvers, MA, USA). CFSE (Cat# C34554) and Fluo-4 AM (Cat# F14201) were bought from Thermo Fisher Scientific (Ottawa, ON, Canada). BAPTA-AM (Cat# HY-100545) was purchased from MCE (Monmouth Junction, NJ, USA).

### 2.3. Cell Lines

Mouse cancer cell lines YAC-1, NS-1 and 4T-1 were obtained from ATCC. YAC-1 and 4T-1 cells were cultured in RPMI-1640 (Gibco, Cat# C11875500) with 10% fetal bovine serum (FBS), whereas NS-1 cells were cultured in DMEM (Cytiva, Cat# SH30022.01) with 10% FBS. All cells were frozen at passages 2 to 5 after purchase. Experiments were performed using passages 3 to 15 after removal from liquid nitrogen. Mycoplasma testing was performed monthly. Cell line authentication was performed by ATCC.

### 2.4. Isolation of NK Cells

Isolation of the mouse splenic NK cells was performed using the EasySep Mouse NK Cell Isolation Kit (Cat# 19855, StemCell Technologies, Vancouver, BC, Canada). Isolated NK cells were incubated in 1% FBS-RPMI-1640 at 37 °C in 5% CO_2_.

### 2.5. Giemsa Staining

Isolated NK cells were fixed with 4% paraformaldehyde, stained with Giemsa solution and observed under an optical microscope.

### 2.6. Flow Cytometry

Balb/c mouse spleens were dissected and mechanically disrupted to form a single-cell suspension. Cells were washed with PBS and then stained with PE-conjugated anti-mouse CD49b and FITC-conjugated anti-mouse NKp46 antibodies for 30 min. Cells were measured on a Guava EasyCyte HT (Merck KGaA, Darmstadt, Germany), and data were processed using FlowJo 10 software (FlowJo LLC, Ashland, OR, USA).

### 2.7. CCK-8 Cell Viability Assay

Isolated NK cells (2 × 10^4^ cells per well) in a 96-well plate were incubated in 1% FBS-RPMI-1640 containing 0–10 ng/mL activin A or 20 ng/mL IL-2 at 37 °C in 5% CO_2_ for 24 h. Each well was replaced with 100 μL of fresh medium supplemented with 10 μL of CCK-8 reagent and incubated at 37 °C for 2 h. The absorbance was determined at 450 nm (A450 nm) using a microplate spectrophotometer (Gene Company Limited, Shanghai, China). Each experiment was carried out in triplicate.

### 2.8. NK Cell Cytotoxicity Assay

Isolated NK cells (1 × 10^5^ cells/well) in a 24-well plate were incubated in 1% FBS-RPMI-1640 containing 10 ng/mL activin A, 20 ng/mL IL-2 or 10 ng/mL activin A plus 20 ng/mL IL-2 at 37 °C in 5% CO_2_ for 24 h and then co-cultured with CFSE-labeled YAC-1 cells (1 × 10^4^ cells/well) at an effector/target ratio of 10:1 for 4 h. Then, cells were stained with 1 μL PI at room temperature (RT) for 5 min in darkness and analyzed by flow cytometry. The killing rate of NK cells to YAC-1 cells was determined using the following equation: killing rate = (PI^+^ CFSE-labeled YAC-1 cells/all CFSE-labeled YAC-1 cells) × 100%.

### 2.9. Transwell Chamber Cell Migration Assay

First, 1 × 10^5^ NK cells were seeded into the upper chamber of each 24-well transwell filter (pore size, 5 μm; diameter insert, 6.5 mm; Costar Corporation; Cat# CLS3421) with 0.2 mL 1% FBS-RPMI-1640, and the lower chamber was filled with 1% FBS-RPMI-1640 containing 100 ng/mL CXCL12, 10 ng/mL activin A or 100 ng/mL CXCL12 plus 10 ng/mL activin A. After 4 h at 37 °C, the number of cells migrated was quantified using the Guava EasyCyte HT (Merck KGaA, Darmstadt, Germany).

### 2.10. Microfluidic Cell Migration Assay

A microfluidic device was prepared by the standard photolithography and soft-lithography techniques described previously [22]. Before the experiment, the microfluidic chip was coated with fibronectin (30 μg/mL) for 2 h and then incubated in 0.4% bovine serum albumin (BSA) diluted by RPMI-1640 for 30 min at 37 °C. Through the cell inlets of the microfluidic device, the cells were loaded to the parallel test units and allowed to align in the docking structures. An equal volume of RPMI-1640 medium and chemoattractant solution were added into the pairs of source wells for configuring different gradient conditions. The chemoattractant solutions included 200 ng/mL CXCL12, 20 ng/mL activin A or 200 ng/mL CXCL12 plus 20 ng/mL activin A. The device was kept at 37 °C. Cell migration images were captured every 15 s for 30 min. Individual cell movement in time-lapse images was tracked and analyzed by the “Manual Tracking” plug-in NIH ImageJ. As soon as a cell entered a migration channel with more than one cell length, it was defined as a migrating cell. Twenty migrating cells were tracked and analyzed in each experiment. Experiments were performed in triplicates for each condition. Migration parameters, including accumulated distance (AD), Euclidean distance (ED), cell speed and chemotactic index (CI) [5,22], were quantified (see Supplementary Figure S1).

### 2.11. Transwell Chamber Cell Invasion Assay

Cell invasion was assessed using a transwell chamber pre-coated with 20 μg Matrigel. In brief, 1 × 10^5^ NK cells were seeded into the upper chamber coated with Matrigel, and the lower chamber was filled with 1% FBS-RPMI-1640 containing 100 ng/mL CXCL12, 10 ng/mL activin A or 100 ng/mL CXCL12 plus 10 ng/mL activin A. After 4 h at 37 °C, the number of cells invaded was analyzed with Guava EasyCyte HT (Merck KGaA, Darmstadt, Germany).

### 2.12. Immunofluorescent Staining

In the microfluidic migration experiment, NK cells were immobilized with 4% paraformaldehyde at 10 min after the addition of chemoattractant solutions. Thirty minutes later, the NK cells were washed once with PBS, followed by the addition of 0.3% Triton-100 to induce cell membrane disruption for 20 min. Subsequently, the NK cells were subjected to three washes of 5 min each with PBS. Following this, a concentration of 10 μg/mL phalloidin, known for its strong affinity to filamentous actin (F-actin), was added and incubated overnight at 4 °C Ultimately, the NK cells were stained with DAPI for 10 min, followed by three additional washes with PBS. Fluorescence imaging was performed using Zeiss ZEN 2.3 blue imaging software (Carl Zeiss, Oberkochen, Germany).

### 2.13. Real-Time Cell Analysis

Initially, 50 μL RPMI-1640 medium was added to each well of an E16 xCELLigence microtiter plate, which was then inserted into the real-time cell analysis (RTCA) analyzer (ACEA Biosciences, San Diego, CA, USA). After the background impedance for each well was measured for 1 min, NK cells (5 × 10^4^ cells) in 100 μL of 1% FBS-RPMI-1640 containing 100 ng/mL CXCL12, 10 ng/mL activin A or 100 ng/mL CXCL12 plus 10 ng/mL activin A were plated into the E-plate and cultured in the RTCA system installed in a CO_2_ incubator for 4 h. Impedance was detected every 5 min and expressed as cell index (CI) by the RTCA-integrated software 2.0 of the xCELLigence system. Each experiment was performed in duplicate and repeated three times.

### 2.14. Western Blotting

Cells were lysed by the protein extraction reagent (Cat# 78503, Thermo Fisher Scientific, Waltham, MA, USA) supplemented with phosphatase and protease inhibitor cocktail (Cat# 1862209, Thermo Fisher Scientific, USA) and 5 mM EDTA solution. Proteins were quantified using the BCA protein assay kit (Cat# 23225, Thermo Fisher Scientific, USA) following the manufacturer’s instructions. Then, 20 μg of protein extracts were separated by SDS–polyacrylamide gel electrophoresis (10% SDS-PAGE Tris-glycine gels) and then transferred onto a polyvinylidene difluoride membrane. The membranes were blocked with 5% BSA-TBS-Tween for 2 h at RT and incubated in primary antibodies overnight at 4 °C. Furthermore, the membranes were incubated in horseradish peroxidase-conjugated secondary antibody for 2 h at RT, followed by ECL detection (GE Healthcare, Buckinghamshire, UK, Cat# 29018903, Cat# 29018904). Finally, the membranes were scanned using the luminescent image analyzer LAS-4000 (Fujifilm, Tokyo, Japan).

### 2.15. Kinase Inhibition

NK cells were seeded into a 12-well plate and pretreated with 0.1% DMSO or 10 μM AKT inhibitor (GSK690693) in 0.1% DMSO for 1 h. Then, the pretreated NK cells were used in the transwell migration assay.

### 2.16. Calcium Flux Assay

NK cells were incubated with 4 mM Fluo-4 AM in 1% FBS-RPMI-1640 at 37 °C for 40 min in the dark and washed with RPMI-1640 twice. Fluo-4-loaded NK cells were incubated for another 30 min in the dark and then divided into different tubes for recording Fluo-4 baseline fluorescence signal (F_0_) for 1 min by flow cytometry. Cells were stimulated with 1% FBS-RPMI-1640 containing 100 ng/mL CXCL12, 10 ng/mL activin A or 10 ng/mL activin A plus 100 ng/mL CXCL12, and the Fluo-4 signal of stimulated cells (F) was recorded for another 3 min. The experiment was repeated at least three times. The kinetics of Fluo-4 signal intensity was analyzed using FlowJo 10 software (FlowJo LLC, Ashland, OR, USA). The Fluo-4 intensity of stimulated cells was normalized to the baseline signal for comparison (F/F_0_).

### 2.17. Calcium Chelation

BAPTA-AM, a cell-permeable chelator, is selective for Ca^2+^ and can be used to control intracellular Ca^2+^ levels. In order to clarify the effect of intracellular Ca^2+^ on mouse NK cell migration, the mouse NK cells were treated with BAPTA-AM. In brief, the mouse NK cells were pretreated with 0.1% DMSO or 10 μM BAPTA-AM in 0.1% DMSO for 10 min, and then the pretreated NK cells were used in the transwell migration assay.

### 2.18. RT-PCR

The total RNA of cells was extracted with TRIzol reagent (Cat# 9108, Takara, Dalian, China) according to the manufacturer’s instructions. With the cDNA Synthesis Kit (Cat# CW2020, Cwbio, Taizhou, China), 1 μg RNA was used for reverse transcription into cDNA. A PCR kit (Cat# CW0690, Cwbio, Taizhou, China) was used to perform PCR under the following conditions: 95 °C for 90 s; followed by 30 cycles at 94 °C for 30 s, 56 °C for 20 s, and 72 °C for 40 s; and a final extension at 72 °C for 10 min. Two percent agarose gel electrophoresis was used to separate PCR products which were stained using Super Gelred (US EVERBRIGHT, Suzhou, China, Cat# S2001). The specific bands were examined by Image J 1.51j8 software, and mRNA levels of target genes were normalized against *GAPDH* levels [11]. The primer sequences are presented in Table 1.

### 2.19. Enzyme-Linked Immunosorbent Assay

The culture supernatants of mouse myeloma cell line NS-1 cells and mouse breast cancer cell line 4T-1 cells were collected, and the levels of activin A in the supernatants were determined by ELISA kits according to the manufacturer’s protocol (R&D).

### 2.20. Establishment of Tumor-Bearing Mice with NS-1 Cells

All procedures were approved by the Animal Ethics Committee of Jilin University. The tumor-bearing experiment was performed as described previously [11]. In brief, the NS-1 cells of mouse myeloma in the logarithmic growth phase were resuspended in saline. Then, NS-1 cells (2 × 10^6^/100 μL saline) were inoculated on the back of Balb/c male mice (6- to 8-week-old) via subcutaneous injection, and then tumor size was detected daily until tumor volume reached ~105 ± 25 mm^3^. The mice were then randomly divided into two groups, which received intratumoral injections of normal saline (1 μL) or activin A (20 ng in 1 μL saline) once every 24 h, three times consecutively. The mice were anesthetized and killed 24 h after the last injection. The percentage of NK cells in tumors was detected using the Guava EasyCyte HT (Merck KGaA, Darmstadt, Germany).

### 2.21. Establishment of Tumor-Bearing Mice with 4T-1 Cells

The 4T-1 cell tumor-bearing mice experiment was performed as above. When tumors grew to ~105 ± 25 mm^3^, the mice were randomly divided into two groups, which received an intratumoral injection of 200 ng IgG or anti-activin A antibody in 1 μL normal saline once every 24 h, three times consecutively. The mice were anesthetized and killed 24 h after the last injection. The percentage of NK cells in the tumor was detected using the Guava EasyCyte HT (Merck KGaA, Darmstadt, Germany).

### 2.22. Immunofluorescence Assay for NK Cells in Tumor Tissue

The tumor tissues from 4T-1 cell-bearing mice were fixed in 4% paraformaldehyde at 4 °C overnight. Dehydration was carried out by incubation in a sucrose gradient, 5% sucrose for 1 h, 10% sucrose for 2 h, 20% sucrose overnight and 30% sucrose overnight until the tumor tissues completely bottomed. The dehydrated tumor tissues were embedded in OCT (SAKURA, Shanghai, China, Cat# 4583), snap-frozen in liquid nitrogen and stored at −80 °C. The embedded tumor tissues were cut into 10 μm continuous sections using a frozen microtome (Leica Biosystems, Nußloch, Germany) and pasted on adhesive slides. After one night, tumor tissues were washed 3 times with PBS for 5 min each time. Slides were blocked for 30 min at RT with 10% donkey serum diluted in PBS and incubated overnight at 4 °C with FITC-labeled anti-CD49b antibody. After three washed with PBS, the slides were stained with DAPI for 10 min. Finally, after three washed with PBS, the slides were sealed with an anti-fluorescence quenching sealing solution. Fluorescence imaging was performed and processed by Zeiss ZEN 2.3 Blue imaging software (Carl Zeiss, Oberkochen, Germany).

### 2.23. Statistical Analysis

Data were presented as mean ± standard error of mean (SEM) or mean ± standard deviation (S.D.). Statistical analysis was performed in GraphPad Prism Software (version 8.0) using one-way ANOVA followed by Tukey’s multiple comparisons test or Student’s *t* test. A difference at *p* < 0.05 was considered to be statistically significant.

## 3. Results

### 3.1. Activin A Does Not Affect the Viability and Cytotoxicity of Mouse NK Cells

**Figure 1:**
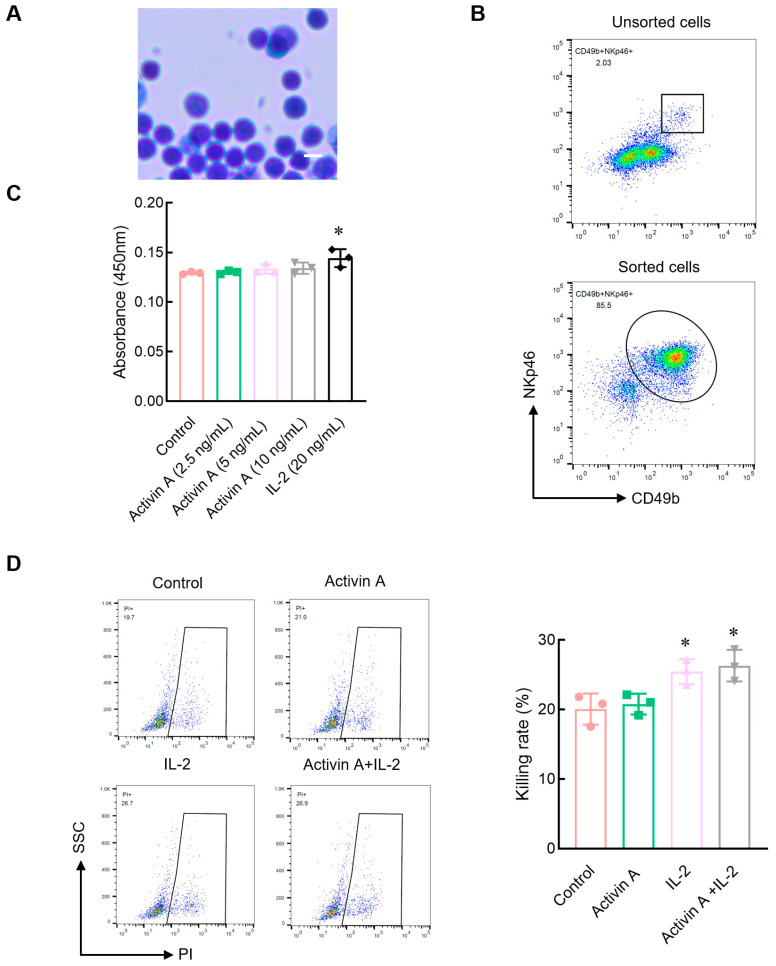
Effect of activin A on viability and cytotoxicity of mouse NK cells. (**A**) Cells sorted by magnetic beads were stained with Giemsa. Scale bar = 10 µm. (**B**) The cells sorted by magnetic beads were identified by flow cytometry with anti-CD49b and NKp46 antibodies. (**C**) The viability of mouse NK cells was examined by CCK-8 assay (*n* = 3). (**D**) The killing rate of NK cells was assayed by flow cytometry, after treatment with the culture medium (Control), 20 ng/mL IL-2, 10 ng/mL activin A or 20 ng/mL IL-2 + 10 ng/mL activin A (*n* = 3). * *p* < 0.05, compared with the control group (mean ± S.D., one-way ANOVA).

Using immunomagnetic beads, mouse NK cells were isolated from the spleen by negative selection. Giemsa staining showed that isolated cells were round in shape with a large oval nucleus on one side and had a uniform and compact nuclear chromatin corresponding to the morphological characteristics of the ILCs (Figure 1A). The flow cytometry results showed that the ratio of CD49b^+^NKp46^+^NK cells was more than 85% (Figure 1B), indicating that isolated cells were suitable for NK cell experiments.

Previous studies have shown that 10 ng/mL of activin A promotes IL-2 production but inhibits IL-9 secretion by NK cells [15]. In this study, the CCK8 assay was used to determine the viability of NK cells treated with 0–10 ng/mL activin A. The results showed that activin A had almost no effect on mouse NK cell viability, while IL-2 as a positive control increased mouse NK cell viability (Figure 1C). NK cells are potent cytotoxic lymphocytes of the innate immune system. Therefore, we examined the cytotoxic activity of mouse NK cells against CFSE-labeled YAC-1 cells. The results showed that compared to the control group, IL-2 significantly increased the cytotoxicity of mouse NK cells, but 10 ng/mL activin A had no significant effect on the cytotoxicity of mouse NK cells. There was also no significant difference between the IL-2-alone group and the IL-2 plus activin A group (Figure 1D).

### 3.2. Activin A Induces Mouse NK Cell Migration

**Figure 2:**
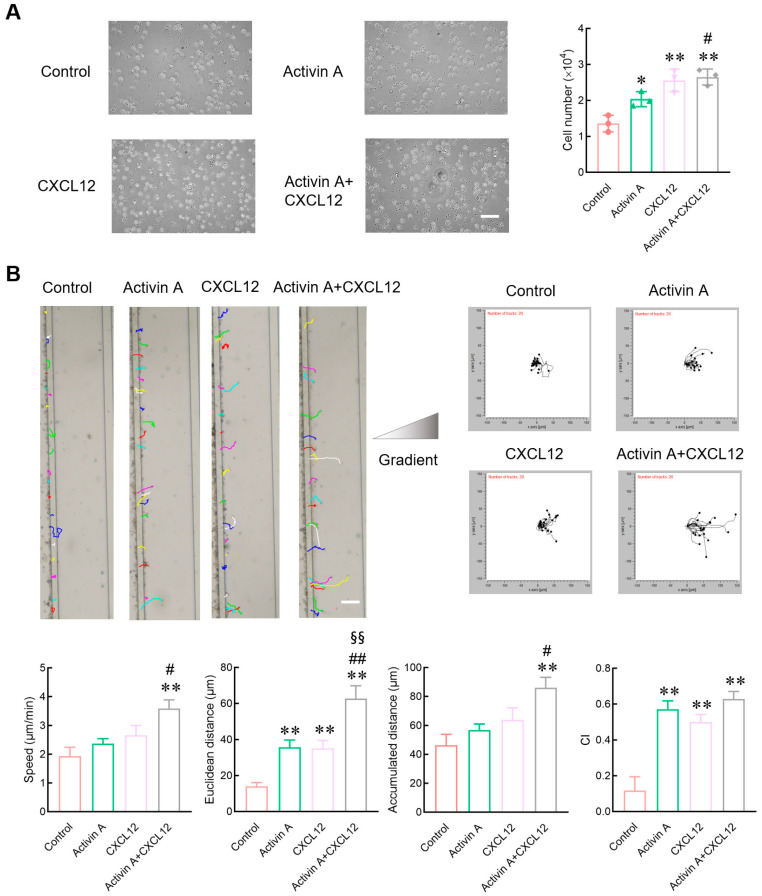
Effect of activin A on the migration of mouse NK cells. (**A**) Transwell assay was used to detect migration of NK cells treated with culture medium (Control), 10 ng/mL activin A, 100 ng/mL CXCL12 or 10 ng/mL activin A + 100 ng/mL CXCL12 (*n* = 3). Scale bar = 30 µm. * *p* < 0.05, ** *p* < 0.01, compared with the control group; # *p* < 0.05, compared with the activin A group (one-way ANOVA; mean ± S.D.). (**B**) NK cell migration was examined by microfluidic chips after treatment with culture medium (Control), 20 ng/mL activin A, 200 ng/mL CXCL12 or 20 ng/mL activin A + 200 ng/mL CXCL12. Data related to cell migration were analyzed by NIH ImageJ software (*n* = 20). Scale bar = 100 µm. ** *p* < 0.01, compared with the control group; # *p* < 0.05, ## *p* < 0.01, compared with the activin A group; §§ *p* < 0.01, compared with the CXCL12 group (one-way ANOVA; mean ± SEM).

Peripheral blood NK cells must migrate to the tumor site to exert their effect. Therefore, a transwell assay was used to determine the effect of activin A on NK cell migration. As a positive control, CXCL12 significantly increased the number of mouse NK cells migrated compared to the control group, consistent with literature reports [19,23], and activin A also increased the number of migrated mouse NK cells (Figure 2A).

Evaluation indices of cell migration include the number of migrated cells and various other parameters such as migration speed, migration distance and migration direction. Microfluidic chips provide suitable environments for real-time measurements of migration direction and speed. In the present study, the microfluidic chip was further used to examine the migration of mouse NK cells. CXCL12 significantly increased the Euclidean distance of mouse NK cells and the chemotaxis index (CI) compared to the control group. Activin A also significantly increased the Euclidean distance and the CI of mouse NK cells, and the combined group of activin A and CXCL12 showed the most potent effect on inducing the migration of NK cells (Figure 2B and Supplementary Figure S2). These results indicate that activin A can induce mouse NK cell migration and has a synergistic effect with CXCL12.

### 3.3. Activin A Increases the Invasion and Inhibits the Adhesion of Mouse NK Cells

**Figure 3:**
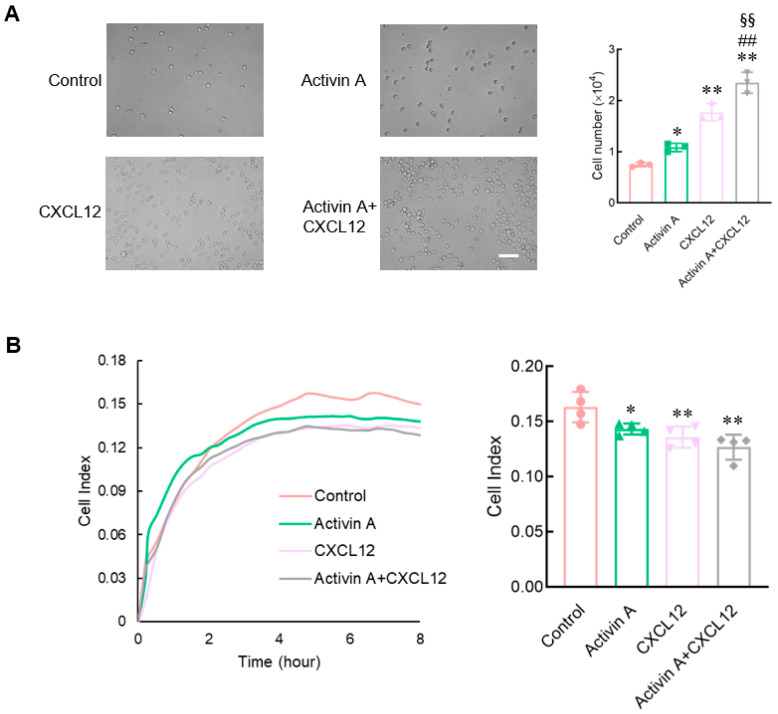
Effects of activin A on the invasion and adhesion of mouse NK cells. (**A**) NK cell invasion was determined by Matrigel-coated transwell assay after treatment with culture medium (Control), 10 ng/mL activin A, 100 ng/mL CXCL12 or 10 ng/mL activin A + 100 ng/mL CXCL12 (*n* = 3). Scale bar = 50 µm. * *p* < 0.05; ** *p* < 0.01, compared with the control group; ## *p* < 0.01, compared with the activin A group; §§ *p* < 0.01, compared with the CXCL12 group (one-way ANOVA; mean ± S.D.). (**B**) Real-time cell adhesion was assessed by RTCA. NK cells were treated with culture medium (Control), 10 ng/mL activin A, 100 ng/mL CXCL12 or 10 ng/mL activin A + 100 ng/mL CXCL12 (*n* = 4). * *p* < 0.05; ** *p* < 0.01, compared with the control group (one-way ANOVA; mean ± S.D.).

NK cells migrate and infiltrate the primary tumor site through the extracellular matrix (ECM). This study used a Matrigel-coated transwell chamber to simulate the ECM and examine the invasion of mouse NK cells. Compared to the control group, CXCL12 as a positive control significantly increased the invasion of NK cells, and activin A also significantly increased the number of invaded NK cells. The combination group of activin A and CXCL12 had the strongest effect on increasing NK cell invasion (Figure 3A).

Cell motility, which is closely related to the changes in cell adhesion, requires a certain degree of adhesion, but excessive adhesion limits cell migration [16,24,25]. This study used real-time cell analysis (RTCA) to detect the adhesion of mouse NK cells. Either CXCL12 or activin A significantly inhibited NK cell adhesion compared to the control group (Figure 3B). The above results suggest that activin A induces mouse NK cell migration by promoting NK cell polarization and inhibiting NK cell adhesion.

### 3.4. Activin A Promotes Polarization of Mouse NK Cells

**Figure 4:**
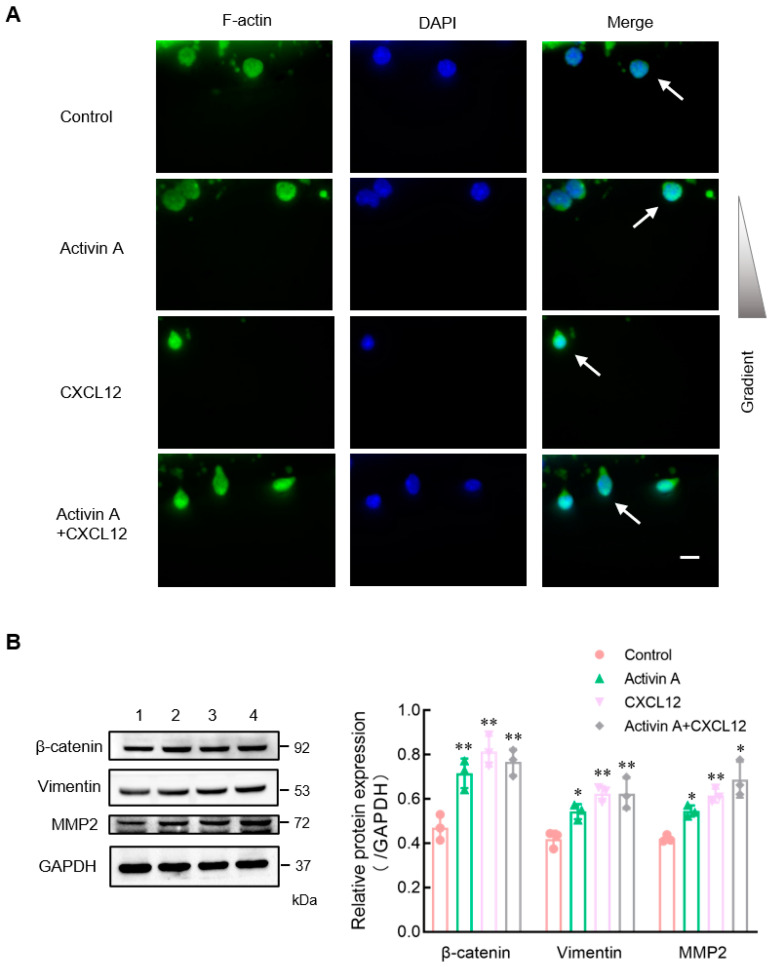
Effects of activin A on F-actin polarization and expression of migration-related proteins of mouse NK cells. (**A**) The nuclei were stained with DAPI (blue) and the cell was stained with FITC-labeled phalloidin that preferentially labels filamentous actin (F-actin) (green). NK cells (white arrows) were treated with culture medium (Control), 20 ng/mL activin A, 200 ng/mL CXCL12 or 20 ng/mL activin A + 200 ng/mL CXCL12. Scale bar = 10 µm. (**B**) Levels of β-catenin, vimentin and MMP2 protein in mouse NK cells were examined by Western blotting, after treating the cells with culture medium (lane 1), 10 ng/mL activin A (lane 2), 100 ng/mL CXCL12 (lane 3) or 10 ng/mL activin A + 100 ng/mL CXCL12 (lane 4) for 2 h. The levels of protein expression were normalized against those of GAPDH (*n* = 3). * *p* < 0.05; ** *p* < 0.01, compared with the control group (one-way ANOVA; mean ± S.D.).

Cell motility is also closely related to the polarization distribution of cytoskeletal proteins [16,24,25]. Phalloidin has a high affinity for actin and can selectively bind filamentous actin (F-actin) without binding monomeric actin (G-actin) [26,27]. This study used green fluorescent phalloidin to examine the expression and distribution of F-actin in mouse NK cells during cell migration in microfluidic chips. The distribution of F-actin of NK cells was uniform in the control group, while it was significantly polarized in the CXCL12 group, the activin A group and the activin A plus CXCL12 group (Figure 4A).

Infiltration, migration and adhesion of NK cells are related to the degradation of the ECM and are affected by various proteins [28]. To further determine the effect of activin A on NK cell migration and invasion, Western blotting was used to examine the expression of migration-related proteins β-catenin, vimentin and matrix metalloproteinase 2 (MMP2) in NK cells. Compared to the control group, the expression of β-catenin and vimentin was enhanced in activin A-treated NK cells, and the expression of MMP2, which is involved in ECM degradation, was also significantly increased (Figure 4B).

### 3.5. Activin A Increases the Levels of p-SMAD3 and p-AKT Proteins in Mouse NK Cells

**Figure 5:**
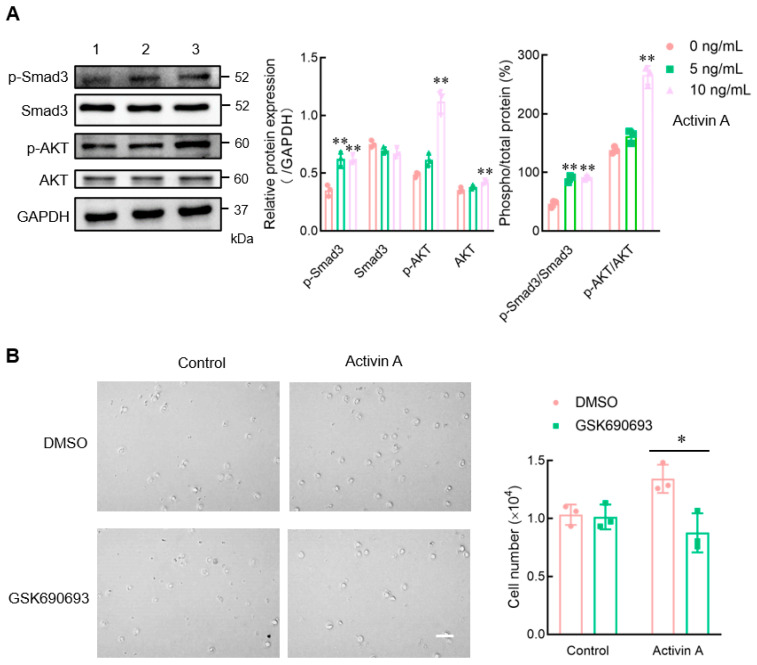
Effect of activin A on the expression of signaling proteins in mouse NK cells. (**A**) Levels of p-Smad3, Smad3, p-AKT, and AKT proteins in mouse NK cells were examined by Western blotting, after treating the cells with culture medium (lane 1), 5 ng/mL activin A (lane 2) or 10 ng/mL activin A (lane 3) for 1 h. The levels of these proteins’ expression were normalized against those of GAPDH (*n* = 3). * *p* < 0.05; ** *p* < 0.01, compared with the control group (one-way ANOVA; mean ± S.D.). (**B**) After pretreating the cells with DMSO or DMSO-dissolved GSK690693 (10 μM) for 1 h, the migration of mouse NK cells was detected by the transwell assay (*n* = 3). Scale bar = 30 µm. * *p* < 0.05, compared with the DMSO group (Student’s *t* test; mean ± S.D.).

Activin A can regulate cell activity through the canonical SMAD or non-SMAD-dependent signaling pathways [21]. In the present study, Western blotting was used to examine the expression of signaling proteins to better understand the signal transduction mechanism of activin A-induced mouse NK cell migration. Activin A significantly increased the p-SMAD3 protein level in mouse NK cells and upregulated the p-SMAD3/SMAD3 ratio, consistent with previous studies [15]. Furthermore, activin A significantly increased the level of p-AKT protein and the p-AKT/AKT ratio in mouse NK cells (Figure 5A). After the cells were pretreated with DMSO-diluted AKT inhibitor (GSK690693) for 1 h, the transwell assay showed that GSK690693 attenuated activin A-induced mouse NK cell migration compared to the DMSO control group (Figure 5B). The above results suggested that activin A regulated mouse NK cell migration through SMAD3 and SMAD-independent AKT signaling.

### 3.6. Activin A Increases Ca^2+^ Levels in Mouse NK Cells

**Figure 6:**
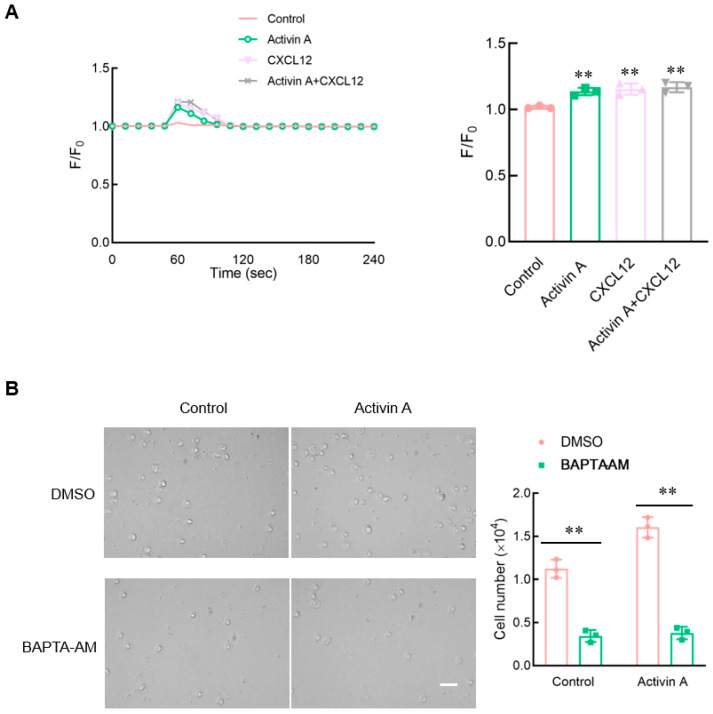
Effect of activin A on calcium flux in mouse NK cells. (**A**) Kinetics of calcium mobilization was assessed using mouse NK cells loaded with Fluo-4 AM, after treating the cells with medium (Control), 10 ng/mL activin A, 100 ng/mL CXCL12 or 10 ng/mL activin A + 100 ng/mL CXCL12. The Ca^2+^ level is represented by the Fluo-4 signal intensity normalized to the baseline (F/F0). The graph shows the peak values of calcium signal upon stimulation under the different treatments (*n* = 3). ** *p* < 0.01, compared with the control group (one-way ANOVA; mean ± S.D.). (**B**). Migration of mouse NK cells pretreated with BAPTA-AM (10 μM) was examined using a transwell assay (*n* = 3). Scale bar = 30 µm. ** *p* < 0.01, compared with the DMSO group (Student’s *t* test; mean ± S.D.).

Calcium signaling has been considered to be an essential factor influencing cell migration [21]. Flow cytometry was used to perform the calcium assay using the Fluo-4 Direct Calcium Assay Kit. Both activin A and CXCL12 significantly increased the intracellular Ca^2+^ levels of mouse NK cells compared to the control group (Figure 6A).

To clarify the effect of calcium influx on mouse NK cell migration, mouse NK cells were pretreated with BAPTA-AM, a Ca^2+^ chelator, and NK cell migration was detected using the transwell assay. The results showed that after BAPTA-AM chelated Ca^2+^ in mouse NK cells, the spontaneous cell migration of mouse NK cells decreased, and activin A-induced mouse NK cell migration was also significantly inhibited (Figure 6B). These results indicated that activin A-induced mouse NK cell migration was associated with increased Ca^2+^ influx.

### 3.7. Exogenous Activin A Increases NK Cell Infiltration into NS-1 Cell Solid Tumors

**Figure 7:**
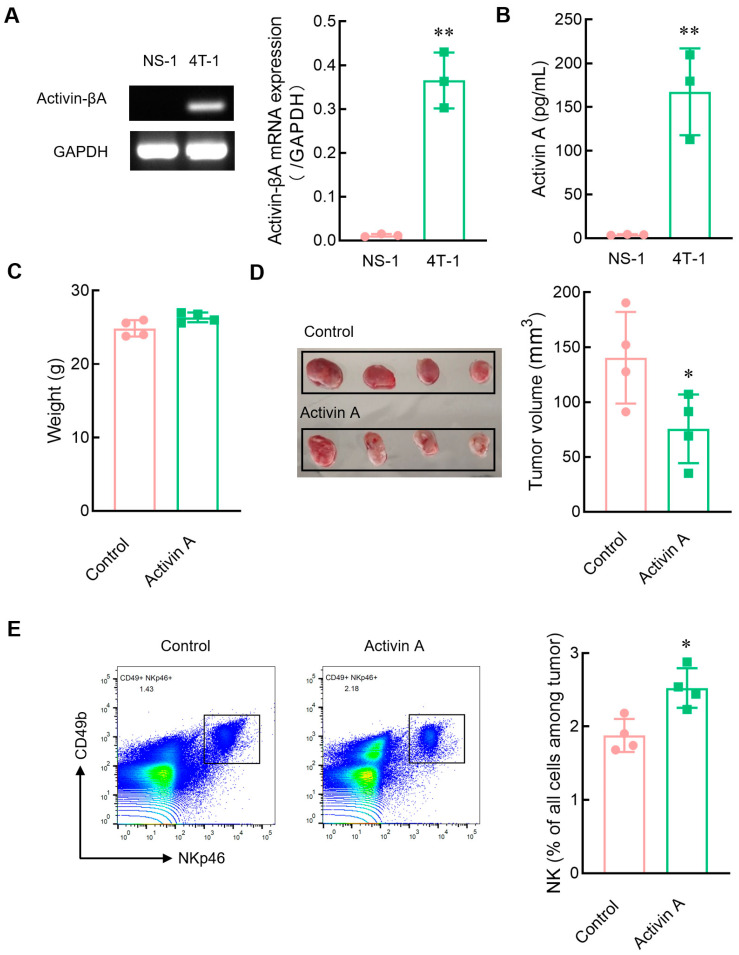
Effects of exogenous activin A on the infiltration of NK cells in NS-1 cell solid tumors. (**A**) The expression of activin-βA mRNA in NS-1 and 4T-1 cells was detected by RT-PCR (*n* = 3). ** *p* < 0.01, compared with the NS-l group (Student’s *t* test; mean ± S.D.). (**B**) The level of activin A protein in the culture supernatants of NS-1 and 4T-1 cells was determined by ELISA (*n* = 3). ** *p* < 0.01, compared with the NS-l group (Student’s *t* test; mean ± S.D.). (**C**–**E**) The NS-1 cell solid tumors were injected with 1 μL normal saline (Control) or 20 ng activin A in 1 μL normal saline every 24 h, three times continuously, and then (**C**) the body weight of mice was recorded, (**D**) tumor volume was measured, (**E**) and the percentage of NK cells infiltrating the tumor was examined by flow cytometry (*n* = 4). * *p* < 0.05, compared with the control group (*n* = 4) (Student’s *t* test; mean ± S.D.).

To further determine the relationship between activin A and NK cell infiltration in tumors, we first analyzed the expression of activin A in tumor cells. Mouse myeloma NS-1 cells hardly secreted activin A, while mouse breast cancer 4T-1 cells highly released activin A (Figure 7A,B). Tumor-bearing mice with mouse myeloma NS-1 cells were established to determine the role of activin A in inducing NK cell migration in vivo. Exogenous activin A was injected into solid tumors, and the same volume of normal saline was injected into solid tumors as a control group. There was no significant difference in body weight between the two groups of mice (Figure 7C). However, compared to the saline control group, activin A injection significantly reduced tumor volume (Figure 7D) and significantly increased the proportion of NK cells infiltrating the tumor (Figure 7E).

### 3.8. Blocking the Endogenous Activin A Reduces the Infiltration of NK Cells into 4T-1 Cell Solid Tumors

**Figure 8:**
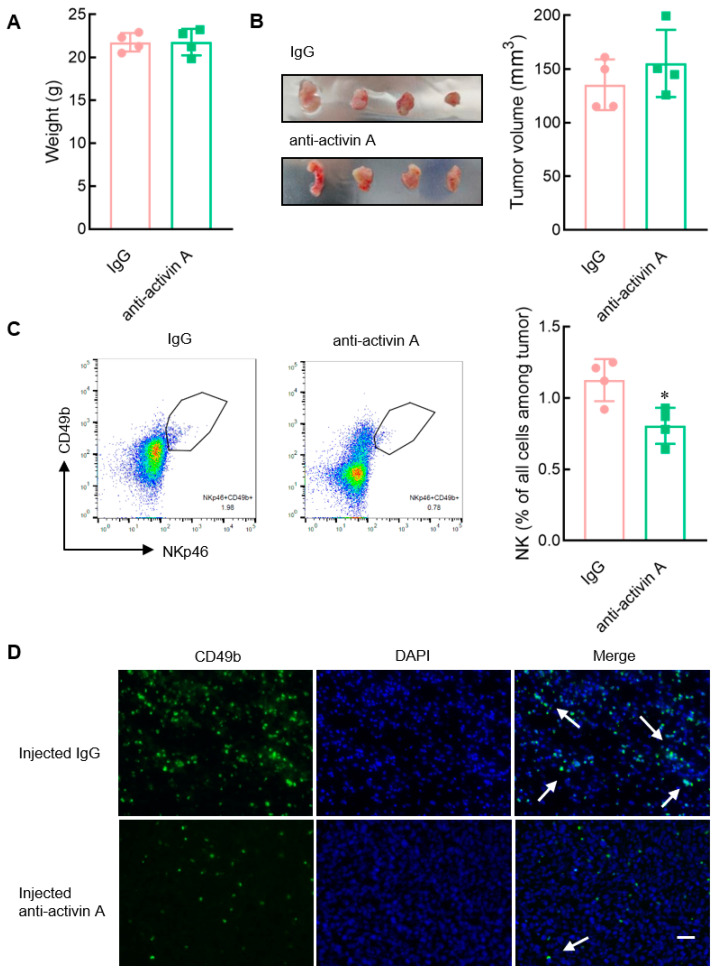
Effect of blocking endogenous activin A on the infiltration of NK cells in 4T-1 cell solid tumors. (**A**–**C**) The 4T-1 cell solid tumors were injected with 200 ng IgG or anti-activin A antibody in 1 μL normal saline every 24 h, three times continuously, and then (**A**) the body weight of mice was recorded, (**B**) tumor volume was measured, (**C**) and the percentage of NK cells infiltrating the tumor was examined by flow cytometry (*n* = 4). * *p* < 0.05, compared with the IgG group (Student’s *t* test; mean ± S.D.). (**D**) The infiltrating NK cells (white arrows) in the tumor tissue were stained with FITC-labeled anti-CD49b antibody (green) and DAPI (blue). Scale bar = 50 µm.

Mouse breast cancer 4T-1 cells highly released activin A (Figure 7). Subsequently, tumor-bearing mice were further established with activin A-secreting 4T-1 cells, and then the effect of endogenous activin A was blocked by injecting anti-activin A antibody into the tumor. Then, the infiltration of NK cells in the tumor was observed. Compared to the control group (normal IgG), there was no significant change in the body weight of the mice in the antibody group (Figure 8A). Although there was a slight increase in tumor volume, it was not statistically significant (Figure 8B). However, in the anti-activin A antibody group, the infiltration of NK cells in the tumor was significantly reduced (Figure 8C).

To visualize NK cell infiltration more intuitively, NK cells in the tumor were examined by anti-CD49b antibody with green fluorescence. The number of NK cells in the tumor was significantly reduced in the anti-activin A antibody group, compared with the control group (Figure 8D), consistent with the above results. These data suggest that the level of activin A in the tumor microenvironment is related to NK cell infiltration.

## 4. Discussion

NK cells are important effector cells for tumor immune surveillance and tumor immunity [7,8] and have potent cytotoxicity to directly kill tumor cells without prior antigen activation through non-MHC-restrictive effects, but circulating NK cells need to migrate to the tumor site to exert their antitumor immune function [5,6]. NK cells can mediate their cytotoxic activity via three distinct pathways. They can release cytotoxic granules containing perforin and granzymes to kill target cells, or they can induce target cell apoptosis by binding to apoptosis death receptors; they can also kill target cells through antibody-dependent cellular cytotoxicity (ADCC) [6]. NK cells are classified as ILC1; they have several features in common (such as producing IFN-γ as their principal cytokine output and killing target cells through ADCC) [29]. Various chemokines and adhesion molecules regulate the chemotactic migration of NK cells toward tumors. For example, CXCL8, CXCL10 and CXCL12 all play a certain regulatory role in NK cell migration [19,23,30]. NK cells also produce many cytokines and chemokines that can activate or help recruit other cells to participate in antitumor responses [19,31]. The tumor microenvironment (TME) refers to the non-cancerous cells (including fibroblasts, endothelial cells, neurons, adipocytes, and immune cells) and their non-cellular components, including the extracellular matrix and soluble products (such as chemokines, cytokines and growth factors) present in tumors [32]. Although some chemokines have been found to induce NK cell migration [19,23,30], there may be many unidentified regulators of NK cell migration in the complex tumor microenvironment.

Activin A, a member of the TGF-β superfamily, is named for its ability to promote follicle-stimulating hormone (FSH) secretion by anterior pituitary cells [10]. Studies have found that activin A is widely distributed in gonadal and extra-gonadal tissues; plays an important role in the inflammatory response, immune regulation, tissue repair, and tumor formation [9,12,21]; and regulates the activities of various immune cells, including neutrophils, monocytes/macrophages, Th9 cells and NK cells [14,15,16,33,34]. Under physiological conditions, the level of activin A in peripheral blood is usually <0.5 ng/mL, whereas in tumors or pregnancy, the level of activin A in peripheral blood can increase up to 10 ng/mL [35,36]. Previous studies have shown that 10 ng/mL of activin A promotes IL-2 secretion by NK cells but inhibits IL-9 production by NK cells, indicating that 10 ng/mL of activin A can affect NK cell activity [15]. Therefore, this study used 0–10 ng/mL activin A to treat mouse NK cells in vitro. We found that activin A had no significant effect on NK cell viability and cytotoxicity. However, not only did CXCL12, a chemokine used as a positive control, induce chemotactic migration of mouse NK cells, but activin A also significantly induced the migration of mouse NK cells.

The binding of chemokines to receptors causes conformational changes and triggers intracellular signals that drive cell polarization, migration, adhesion and homing [30,37]. Many studies have reported that CXCL12 induces NK cell migration [23]. Therefore, CXCL12 was used as the positive control in this study. The transwell assay, which is commonly used to detect cell migration, assesses the ability of cell migration primarily by detecting the number of migrating cells. Resultantly, the effect of cytokines on the migration speed, migration distance and CI of individual cells cannot be determined [21,22]. Therefore, in the present study, we used a microfluidic chip to analyze the NK cell migration process. The results revealed that activin A increased the number of mouse NK cells that migrated and significantly increased the migration distance and CI. Furthermore, activin A and CXCL12 had a synergistic effect in inducing NK cell migration.

Adhesion alteration and cytoskeleton rearrangement are essential factors affecting immune cell migration. Chemokine binding triggers fine cytoskeleton remodeling and supports immune cell migration [38,39]. Actin polymerization and rearrangement control dynamic changes in cell shape during migration, and actin accumulation during migration occurs at the front edge of lymphocytes [40]. Studies have shown that chemokine CXCL12 plays a crucial role in regulating the polarity and migration of T lymphocytes through the polymerization and recombination of the F-actin cytoskeleton [41]. It has also been shown that the rearrangement of the actin cytoskeleton is crucial for NK cell migration [42,43]. In the present study, we found that a polarized distribution of F-actin on one side of NK cells appeared in the CXCL12 group, and a similar polarized distribution was observed in the activin A group. Additionally, activin A significantly inhibited the adhesion of mouse NK cells. These results suggest that activin A-induced migration of mouse NK cells is associated with cytoskeleton remodeling and weakened cell adhesion.

The antitumor function of NK cells depends mainly on their ability to migrate out of the circulation and reach tumor tissue through the extracellular matrix (ECM). The ECM comprises collagen, elastin, proteoglycan and aminoglycan. Matrix metalloproteinases (MMPs) are the main enzymes responsible for the degradation [28]. Changes in cell behavior and morphology are also associated with the expression of various proteins, among which β-catenin and cadherin play a key role in cell adhesion and migration [44,45]. Previous studies have shown that in NK/T cell lymphoma cell lines, it is possible to reduce the level of β-catenin protein and upregulate the E-cadherin protein level to inhibit cell migration [46]. In this study, we found that activin A upregulated the expression of β-catenin and MMP2 proteins in mouse NK cells. At the same time, activin A significantly increased the number of mouse NK cells invaded through Matrigel. These data indicate that activin A may promote the degradation of ECM, which is more favorable for the infiltration of NK cells.

Activin A can regulate cell functions through canonical SMAD and non-canonical signaling pathways. SMAD1/5 and SMAD4 mediate bone morphogenetic protein (BMP) signaling in the canonical SMAD signaling pathway, while SMAD2/3 binds to SMAD4 to transmit activin A/TGF-β signaling [12]. Activin A binds to ActRII to recruit and undergo ActRI phosphorylation and activation. Then, ActRI phosphorylates SMAD2 and SMAD3 to form the heterogeneous complex SMAD2/3 that can bind to SMAD4, which is translocated to the nucleus and drives downstream transcriptional targets [11,12,46]. In addition to the SMAD-dependent pathway, activin A also regulates cell activity through other pathways. For example, activin A stimulates cell migration and invasion of many cells by activating the MAPK and AKT signaling pathways [47]. The present study confirmed that activin A significantly increased the p-SMAD3 level in mouse NK cells and upregulated the p-SMAD3/SMAD3 ratio, similar to the results reported in previous studies [15]. Interestingly, this study also revealed that activin A significantly increased p-AKT levels and upregulated the p-AKT/AKT ratio in mouse NK cells. GSK690693 is a practical, selective, and ATP-competitive pan-AKT kinase inhibitor [48] that blocks the AKT/ZEB1/E-cadherin/vimentin pathway [49]. In this study, GSK690693 pretreatment significantly attenuated activin A-induced mouse NK cell migration. These results suggest that activin A may regulate mouse NK cell migration through AKT signaling in addition to the SMAD3-dependent pathway.

Multiple signaling pathways contribute to the regulation of cell activation and migration. As an essential second messenger in cells, Ca^2+^ regulates various biological activities of cells [21], including cell proliferation and migration. Several studies have shown that during migration, Ca^2+^ pulses preferentially occur at the front of the cells’ forward direction, which may alter cell adhesion and regulate cytoskeleton remodeling, thus supporting cell migration [50]. BAPTA-AM, a Ca^2+^ chelator, inhibits calcium channels and reduces intracellular Ca^2+^ levels, which can significantly reduce IGF-induced migration, invasion, and wound healing of ovarian cancer cells [51]. Previous studies have reported that activin A increases Ca^2+^ influx to induce fibroblast migration [21]. This study found that activin A and CXCL12 significantly increased Ca^2+^ influx in mouse NK cells. In the presence of Ca^2+^ chelator BAPTA-AM, the chemotactic migration of mouse NK cells to activin A was significantly inhibited. These results suggest that Ca^2+^ signaling is involved in regulating mouse NK cell migration by activin A.

NK cells are pivotal innate immune cells in the tumor microenvironment and can kill tumor cells without prior sensitization, playing a key role in tumor immunity [30]. NK cell infiltration into the tumor is crucial for NK cells’ antitumor effects and is also very meaningful for tumor occurrence and development [52]. Many chemokines in the tumor microenvironment are related to effective tumor infiltration of immune cells [30]. Many cells in the tumor microenvironment, such as fibroblasts and monocytes/macrophages, can secrete activin A [53]. Activin A is also highly expressed in various tumor microenvironments [9]. Our data revealed that activin A induces NK cell migration in vitro, but it remains to be determined whether activin A might also play a role in the infiltration of NK cells into the tumor microenvironment in vivo. Therefore, we established a solid tumor model of mouse myeloma NS-1 cells with low activin A secretion and injected saline or activin A into the tumor to observe the infiltration of NK cells. We found that the injection of exogenous activin A significantly increased the number of NK cells that infiltrate tumors. Moreover, a solid tumor model of mouse breast cancer 4T-1 cells with high secretion of activin A was further developed. After blocking endogenous activin A action in tumors with an anti-activin A antibody, we found that the number of tumor-infiltrating NK cells was reduced. Based on the above data, although activin A does not directly enhance the cytotoxicity of NK cells, it may exert an antitumor action by inducing NK cells to infiltrate the tumor, which can be blocked by the anti-activin A antibody.

In this study, microfluidic technology combined with a transwell assay was used to investigate mouse NK cell migration, ensuring the accuracy and intuitiveness of the research results. It was found that activin A, a novel chemokine, induced mouse NK cell migration and tumor infiltration. However, although we conducted research on mouse NK cell migration in vivo through tumor models, our study still has some limitations due to the limited types of tumors and the need for studies on human tumor samples. For example, human lung, liver, breast and other tumors have multiple cell types. Thus, human tumor samples are still needed to investigate the role of activin A in inducing NK cell migration in various cell types of tumors for guiding individualized tumor therapy.

## 5. Conclusions

Previous studies have reported that activin A is highly expressed in tumor tissues [9,53]. In this study, our data suggest that activin A acts as a novel chemokine to induce mouse NK cell migration, which is related to AKT signaling and calcium signaling. Additionally, although activin A can not directly enhance NK cell cytotoxicity, it may enhance the antitumor effect of NK cells by increasing NK cell infiltration into tumor tissues. Therefore, activin A may be used as an adjuvant therapy for tumors.

## Figures and Tables

**Table 1:** Primer sequences for RT-PCR.

Gene	Primer	Sequence (5′-3′)	Fragment Size (bp)	Tm (°C)
*GAPDH*	F	GATTGTTGCCATCAACGACC	372	56
	R	GTGCAGGATGCATTGCTGAC		
*Activin-βA*	F	CGGGTATGTGGAGATAGAGGA	148	56
	R	CAGGTCACTGCCTTCCTTGGA		

## Data Availability

All primary data are available upon reasonable request.

## Supplementary Materials

Quantitative parameters of NK cell migration and NK cells’ images in microfluidic chips can be downloaded at: https://www.mdpi.com/article/10.3390/cells13090728/s1, Figure S1: Quantitative parameters of cell migration; Figure S2: NK cell in microfluidic chips.

## References

[1] Azgomi M.S., Badami G.D., Pizzo M.L., Tamburini B., Dieli C., Manna M.P.L., Francesco Dieli F., Caccamo N. (2024). Integrated Analysis of Single-Cell and Bulk RNA Sequencing Data Reveals Memory-like NK Cell Subset Associated with Mycobacterium tuberculosis Latency. Cells.

[2] Yamamoto Y., Miyazato K., Takahashi K., Yoshimura N., Tahara H., Hayakawa Y. (2018). Lung-resident natural killer cells control pulmonary tumor growth in mice. Cancer Sci..

[3] Rahman S.A., Billingsley J.M., Sharma A.A., Styles T.M., Govindaraj S., Shanmugasundaram U., Babu H., Riberio S.P., Ali S.A., Tharp G.K. (2022). Lymph node CXCR5+ NK cells associate with control of chronic SHIV infection. JCI Insight.

[4] Mahmood S., Nandagopal S., Sow I., Lin F., Kung S.K. (2014). Microfluidic-based, live-cell analysis allows assessment of NK-cell migration in response to crosstalk with dendritic cells. Eur. J. Immunol..

[5] Prager I., Watzl C. (2019). Mechanisms of natural killer cell-mediated cellular cytotoxicity. J. Leukoc. Biol..

[6] Myers J.A., Miller J.S. (2021). Exploring the NK cell platform for cancer immunotherapy. Nat. Rev. Clin. Oncol..

[7] Ma S., Tang T., Wu X., Mansour A.G., Lu T., Zhang J., Wang L.S., Caligiuri M.A., Yu J. (2022). PDGF-D-PDGFRβ signaling enhances IL-15-mediated human natural killer cell survival. Proc. Natl. Acad. Sci. USA.

[8] Barrow A.D., Edeling M.A., Trifonov V., Luo J., Goyal P., Bohl B., Bando J.K., Kim A.H., Walker J., Andahazy M. (2018). Natural killer cells control tumor growth by sensing a growth factor. Cell.

[9] Bloise E., Ciarmela P., Dela Cruz C., Luisi S., Petraglia F., Reis F.M. (2019). Activin A in Mammalian Physiology. Physiol. Rev..

[10] Ma C., Liu Z., Shang S., Jiang L., Lv X., Qi Y., Cui X., Ge J. (2019). Activin A regulates activities of peripheral blood natural killer cells of mouse in an autocrine and paracrine manner. Exp. Cell Res..

[11] Zhang F., Qi Y., Li J., Liu B., Liu Z., Cui X. (2024). Activin A induces apoptosis of human lung adenocarcinoma A549 cells through endoplasmic reticulum stress pathway. Oncol. Rep..

[12] Jiang L., Liu B., Qi Y., Zhu L., Cui X., Liu Z. (2020). Antagonistic effects of activin A and TNF-α on the activation of L929 fibroblast cells via Smad3-independent signaling. Sci. Rep..

[13] Seeger P., Bosisio D., Parolini S., Badolato R., Gismondi A., Santoni A., Sozzani S. (2014). Activin A as a mediator of NK-dendritic cell functional interactions. J. Immunol..

[14] Qi Y., Jiang L., Wu C., Li J., Wang H., Wang S., Chen X., Cui X., Liu Z. (2021). Activin A impairs ActRIIA(+) neutrophil recruitment into infected skin of mice. iScience.

[15] Ma C., Qi Y., Liu H., Wu C., Cui X., Liu Z. (2020). Inhibitory effect of activin A on IL-9 production by mouse NK cells through Smad3 signaling. Biol. Chem..

[16] Wang Y., Qi Y., Qi J., Wu J., Lin F., Cui X., Ge J., Liu Z. (2022). Activin A is a novel chemoattractant for migration of microglial BV2 cells. J. Neuroimmunol..

[17] Pinjusic K., Dubey O.A., Egorova O., Nassiri S., Meylan E., Faget J., Constam D.B. (2022). Activin-A impairs CD8 T cell-mediated immunity and immune checkpoint therapy response in melanoma. J. Immunother. Cancer.

[18] Li N., Cui X., Ge J., Li J., Niu L., Liu H., Qi Y., Liu Z., Wang Y. (2013). Activin A inhibits activities of lipopolysaccharide-activated macrophages via TLR4, not of TLR2. Biochem. Biophys. Res. Commun..

[19] Chen S., Tang W., Yu G., Tang Z., Liu E. (2023). CXCL12/CXCR4 Axis is Involved in the Recruitment of NK Cells by HMGB1 Contributing to Persistent Airway Inflammation and AHR During the Late Stage of RSV Infection. J. Microbiol..

[20] Xie D., Liu Z., Wu J., Feng W., Yang K., Deng J., Tian G., Santos S., Cui X., Lin F. (2017). The effects of activin A on the migration of human breast cancer cells and neutrophils and their migratory interaction. Exp. Cell Res..

[21] Jiang L., Qi Y., Kong X., Wang R., Qi J., Lin F., Cui X., Liu Z. (2021). Activin A as a Novel Chemokine Induces Migration of L929 Fibroblasts by ERK Signaling in Microfluidic Devices. Front. Cell Dev. Biol..

[22] Yang K., Yang X., Gao C., Hua C., Hong C., Zhu L. (2021). A Novel Microfluidic Device for the Neutrophil Functional Phenotype Analysis: Effects of Glucose and Its Derivatives AGEs. Micromachines.

[23] Noda M., Omatsu Y., Sugiyama T., Oishi S., Fujii N., Nagasawa T. (2011). CXCL12-CXCR4 chemokine signaling is essential for NK-cell development in adult mice. Blood.

[24] Paluch E.K., Aspalter I.M., Sixt M. (2016). Focal Adhesion-Independent Cell Migration. Annu. Rev. Cell Dev. Biol..

[25] Sannigrahi M.K., Srinivas C.S., Deokate N., Rakshit S. (2019). The strong propensity of Cadherin-23 for aggregation inhibits cell migration. Mol. Oncol..

[26] Wang J., Guo Y., Xu D., Cui J., Wang Y., Su Y., Liu Y., Shen Y., Jing X., Bai W. (2022). The immunolocalization of cluster of differentiation 31, phalloidin and alpha smooth muscle actin on vascular network of normal and ischemic rat brain. Sci. Rep..

[27] Mazloom-Farsibaf H., Farzam F., Fazel M., Wester M.J., Meddens M.B.M., Lidke K.A. (2021). Comparing lifeact and phalloidin for super-resolution imaging of actin in fixed cells. PLoS ONE.

[28] Edsparr K., Johansson B.R., Goldfarb R.H., Basse P.H., Nannmark U., Speetjens F.M., Kuppen P.J., Lennernäs B., Albertsson P. (2009). Human NK cell lines migrate differentially in vitro related to matrix interaction and MMP expression. Immunol. Cell Biol..

[29] Vivier E., Artis D., Colonna M., Diefenbach A., Di Santo J.P., Eberl G., Koyasu S., Locksley R.M., McKenzie A.N.J., Mebius R.E. (2018). Innate Lymphoid Cells: 10 Years On. Cell.

[30] Wennerberg E., Kremer V., Childs R., Lundqvist A. (2015). CXCL10-induced migration of adoptively transferred human natural killer cells toward solid tumors causes regression of tumor growth in vivo. Cancer Immunol. Immunother..

[31] Bernardini G., Gismondi A., Santoni A. (2012). Chemokines and NK cells: Regulators of development, trafficking and functions. Immunol. Lett..

[32] Xiao Y., Yu D. (2021). Tumor microenvironment as a therapeutic target in cancer. Pharmacol. Ther..

[33] Morianos I., Papadopoulou G., Semitekolou M., Xanthou G. (2019). Activin-A in the regulation of immunity in health and disease. J. Autoimmun..

[34] Robson N.C., Wei H., McAlpine T., Kirkpatrick N., Cebon J., Maraskovsky E. (2009). Activin-A attenuates several human natural killer cell functions. Blood.

[35] Reddy A., Suri S., Sargent I.L., Redman C.W., Muttukrishna S. (2009). Maternal circulating levels of activin A, inhibin A, sFlt-1 and endoglin at parturition in normal pregnancy and pre-eclampsia. PLoS ONE.

[36] Shahul S., Ramadan H., Nizamuddin J., Mueller A., Patel V., Dreixler J., Tung A., Lang R.M., Weinert L., Nasim R. (2018). Activin A and Late Postpartum Cardiac Dysfunction Among Women With Hypertensive Disorders of Pregnancy. Hypertension.

[37] Kremer V., Ligtenberg M.A., Zendehdel R., Seitz C., Duivenvoorden A., Wennerberg E., Colón E., Scherman-Plogell A.H., Lundqvist A. (2017). Genetic engineering of human NK cells to express CXCR2 improves migration to renal cell carcinoma. J. Immunother. Cancer.

[38] de Boer L.L., Vanes L., Melgrati S., Biggs O’May J., Hayward D., Driscoll P.C., Day J., Griffiths A., Magueta R., Morrell A. (2023). T cell migration requires ion and water influx to regulate actin polymerization. Nat. Commun..

[39] Seetharaman S., Etienne-Manneville S. (2020). Cytoskeletal Crosstalk in Cell Migration. Trends Cell Biol..

[40] Ramírez-Santiago G., Robles-Valero J., Morlino G., Cruz-Adalia A., Pérez-Martínez M., Zaldivar A., Torres-Torresano M., Chichón F.J., Sorrentino A., Pereiro E. (2016). Clathrin regulates lymphocyte migration by driving actin accumulation at the cellular leading edge. Eur. J. Immunol..

[41] Freeley M., O’Dowd F., Paul T., Kashanin D., Davies A., Kelleher D., Long A. (2012). L-plastin regulates polarization and migration in chemokine-stimulated human T lymphocytes. J. Immunol..

[42] Fionda C., Stabile H., Molfetta R., Kosta A., Peruzzi G., Ruggeri S., Zingoni A., Capuano C., Soriani A., Paolini R. (2021). Cereblon regulates NK cell cytotoxicity and migration via Rac1 activation. Eur. J. Immunol..

[43] Nieto M., Navarro F., Perez-Villar J.J., del Pozo M.A., González-Amaro R., Mellado M., Frade J.M., Martínez A.C., López-Botet M., Sánchez-Madrid F. (1998). Roles of chemokines and receptor polarization in NK-target cell interactions. J. Immunol..

[44] Zhang T., Liu S., Yang P., Han C., Wang J., Liu J., Han Y., Yu Y., Cao X. (2009). Fibronectin maintains survival of mouse natural killer (NK) cells via CD11b/Src/beta-catenin pathway. Blood.

[45] Wang J., Yan H.B., Zhang Q., Liu W.Y., Jiang Y.H., Peng G., Wu F.Z., Liu X., Yang P.Y., Liu F. (2021). Enhancement of E-cadherin expression and processing and driving of cancer cell metastasis by ARID1A deficiency. Oncogene.

[46] Sugatani T. (2018). Systemic Activation of Activin A Signaling Causes Chronic Kidney Disease-Mineral Bone Disorder. Int. J. Mol. Sci..

[47] Dean M., Davis D.A., Burdette J.E. (2017). Activin A stimulates migration of the fallopian tube epithelium, an origin of high-grade serous ovarian cancer, through non-canonical signaling. Cancer Lett..

[48] Levy D.S., Kahana J.A., Kumar R. (2009). AKT inhibitor, GSK690693, induces growth inhibition and apoptosis in acute lymphoblastic leukemia cell lines. Blood.

[49] Chen W., Wu S., Zhang G., Wang W., Shi Y. (2013). Effect of AKT inhibition on epithelial-mesenchymal transition and ZEB1-potentiated radiotherapy in nasopharyngeal carcinoma. Oncol. Lett..

[50] Hammad A.S., Machaca K. (2021). Store Operated Calcium Entry in Cell Migration and Cancer Metastasis. Cells.

[51] Liu L., Wu N., Wang Y., Zhang X., Xia B., Tang J., Cai J., Zhao Z., Liao Q., Wang J. (2019). TRPM7 promotes the epithelial-mesenchymal transition in ovarian cancer through the calcium-related PI3K/AKT oncogenic signaling. J. Exp. Clin. Cancer Res..

[52] Cózar B., Greppi M., Carpentier S., Narni-Mancinelli E., Chiossone L., Vivier E. (2021). Tumor-Infiltrating Natural Killer Cells. Cancer Discov..

[53] Zessner-Spitzenberg J., Thomas A.L., Krett N.L., Jung B. (2019). TGFβ and activin A in the tumor microenvironment in colorectal cancer. Gene Rep..

