# Supplementary material for: Activin A, a Novel Chemokine, Induces Mouse NK Cell Migration via AKT and Calcium Signaling
Source: Cells. 2024 Apr 23;13(9):728. doi: 10.3390/cells13090728 (PMC11083611; doi:10.3390/cells13090728)
Supplement: Supplementary file 1 [file cells-13-00728-s001.zip › cells-2923493-supplementary.pdf]

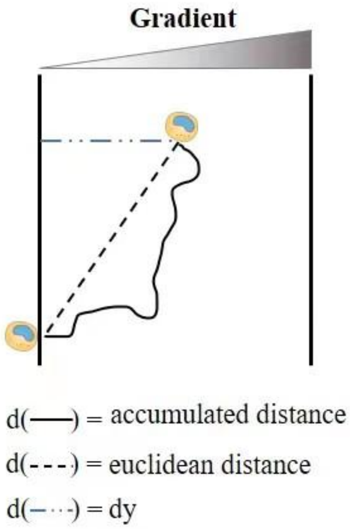

Figure S1. Quantitative parameters of cell migration. The solid black line represents track of migrating NK cell. dy, migration distance to the gradient; the chemotactic index was defined as the ratio of the displacement of cells toward the chemokine gradient (dy) to the accumulated distance (AD) using the equation CI = dy/AD. Cell speed = AD/t, where t is the time of travel.

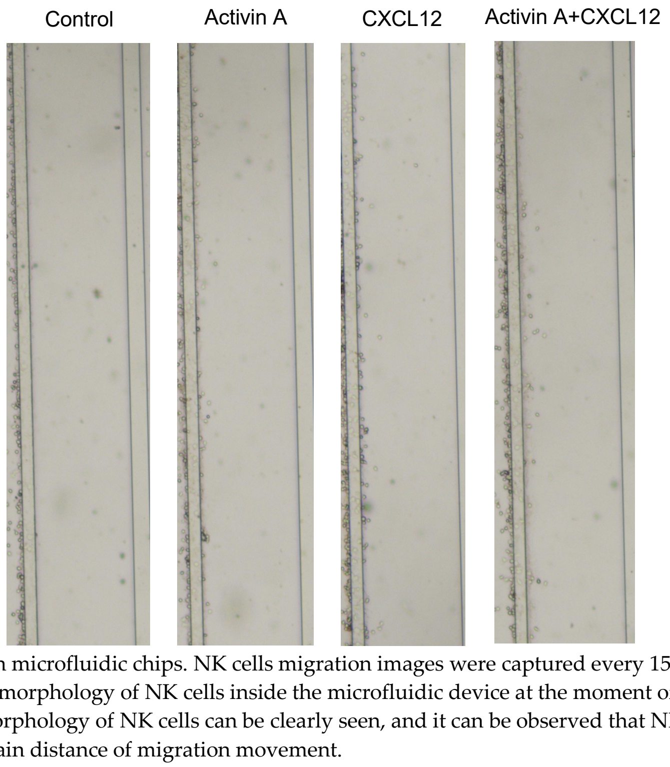

Figure S2. NK cell in microfluidic chips. NK cells migration images were captured every 15 sec for 30 min. The figure shows the morphology of NK cells inside the microfluidic device at the moment of 10 minutes. From the figure, the morphology of NK cells can be clearly seen, and it can be observed that NK cells have undergone a certain distance of migration movement.
